# Caught in the Web of the Net? Part II: A Motivation-Based Developmental Psychopathology Model for the Aberrant Internet Use in (Young) People with Autism Spectrum Disorder

**DOI:** 10.1007/s10567-025-00539-1

**Published:** 2025-07-18

**Authors:** Peter Muris, Henry Otgaar, Franc Donkers, Thomas Ollendick, Anne Deckers

**Affiliations:** 1 Department of Clinical Psychological Science, Faculty of Psychology and Neuroscience, Maastricht University, Maastricht, The Netherlands; 2 Stellenbosch University, Stellenbosch, South Africa; 3 Youz-Parnassia Group, Maastricht, The Netherlands; 4 Catholic University Leuven, Leuven, Belgium; 5 Virginia Polytechnic Institute and State University, Blacksburg, USA; 6 Zuyderland Medisch Centrum, Heerlen, The Netherlands

**Keywords:** Autism spectrum disorder (ASD), Problematic internet use (PIU), Social media use, Developmental psychopathology, Motivational analysis

## Abstract

In Part I (Muris et al. in Clinical Child and Family Psychology Review 22:549–561, 2025), we provided meta-analytic evidence showing that individuals with autism spectrum disorder (ASD) or high levels of autistic traits exhibit higher rates of problematic internet use (PIU), but paradoxically have lower levels of social media use compared to typically developing individuals or those with lower levels of autistic traits. The current theoretical article introduces a motivation-based developmental psychopathology model aimed at clarifying the motives behind the atypical internet and social media use observed in people with ASD or with high levels of autistic traits. We argue that excessive online activities, such as gaming and watching videos, can be understood through specific social, coping, and enhancement motives for internet use, which are especially prominent in ASD due to disorder-specific characteristics such as narrow interests and challenges in face-to-face interactions. In contrast, when it comes to social media use, these three motives operate differently, leading individuals with ASD to exhibit lower motivation to engage in online social interactions compared to neurotypical individuals. The current article emphasizes adolescence as a critical developmental period where internet use can easily become maladaptive and explores the role of parents in regulating the online behaviors of young people with ASD. Finally, the clinical implications of the model are briefly discussed.

## Introduction

Autism spectrum disorder (ASD) is a developmental condition linked to irregular brain development and functioning (Lord et al., 2020). It is typically characterized by two main features: (A) ongoing difficulties with social communication and interaction, including challenges in social-emotional exchange, non-verbal communication, and forming relationships; and (B) repetitive and restricted behaviors and interests (RRBIs), which involve repetitive speech or motor actions, strict adherence to routines, intense, narrow interests, and heightened sensory sensitivities (American Psychiatric Association, 2022). Globally, approximately, 1 per 100 people are diagnosed with ASD (Zeidan et al., 2022), with males being more often affected by this neurodevelopmental disorder than females (with a ratio of 3:1; Loomes et al., 2017). Evidence also indicates that the prototypical characteristics of ASD are also present in the general population. Thus, milder but qualitative similar symptoms of social aloofness, unconventional and/or narrow interests, rigidity and desire for sameness, and sensory peculiarities can also be detected in the general population in people who do not meet the clinical threshold for this neurodevelopmental disorder (Sasson et al., 2013a).

People with ASD often face numerous challenges in navigating everyday life. Specifically, due to the prototypical characteristics of the disorder, many of them struggle with establishing and maintaining social contacts (Tobin et al., 2014), face obstacles with following and completing appropriate education (Nuske et al., 2019a, 2019b) or finding and maintaining proper employment (Scott et al., 2019), and have problems with understanding and regulating their emotions (Cibralic et al., 2019). Given all these difficulties and hardships, individuals with autism often experience significant comorbid psychopathology (Joshi et al., 2010) and generally report a lower quality of life (Øverland et al., 2022).

With the emergence of the internet and its many digital applications and platforms, people's lives have gained an additional dimension (Hoehe & Thibaut, 2020). Researchers have increasingly started to examine how individuals with ASD navigate this online and perhaps challenging world (Ophir et al., 2023). Because navigating the internet requires less communicative and interactive skills and can be conducted without exposing one’s behavioral peculiarities, its use may provide a good way to satisfy the needs and interests of people with ASD. Even though individuals high on the autistic spectrum are strongly attracted to the internet and hence might profit from its presumed benefits (McGhee Hassrick et al., 2021), the psychological literature also echoes warnings regarding the potential risks of online digital media use for this specific neurodivergent population. For example, Lane and Radesky (2019) noted that specific features of modern digital media (e.g., lower complexity in social interactions, strong personalized content, predictable and controllable sensory stimulation, highly rewarding activities) could lead to an excessive use of the internet in people with ASD.

Empirical studies have indeed shown that ASD is associated with higher levels of problematic internet use (PIU). In one of the first investigations on this topic, Mazurek and Wenstrup (2013) compared the online gaming behavior of children and adolescents with ASD (*n* = 202) and typically developing siblings (*n* = 179) by means of a parent-administered survey. The results indicated that children with ASD not only spent significantly more time per day on playing video games than their typically developing siblings but also clearly showed more symptoms of pathological gaming behavior on the Problem Video Game Playing Test (King et al., 2011). More specifically, Mazurek and Wenstrup (2013) noted that “the most commonly reported problems were spending more time playing video games than with friends or family, thinking life would be boring without video games, thinking about video games even when not playing, feeling upset when not able to play, looking forward to the next gaming session, and having trouble disengaging or stopping him/herself from playing [and that all of these] were endorsed at significantly higher levels among children with ASD than among typically developing children” (p. 1266), and eventually concluded that “for many children with ASD, video game play can become salient and preoccupying, and can be associated with distress” (p.1266). Other researchers have obtained similar findings (see for a review: Craig et al., 2021), but based on the extant literature (including studies of young people as well as adults), the conclusion is justified that ASD is not only associated with problematic online gaming but also with an excessive use of a broad range of internet-based media (Eltahir et al., 2025; Murray et al., 2022; Normand et al., 2022; Yuan et al., 2024). In our Part I article (Muris et al., 2025), we adopted a meta-analytic approach to substantiate and quantify this conclusion and found an average effect size for the relation between ASD/autistic traits and PIU of 0.26, implying that out of every 100 people with ASD, 63 will display PIU compared to 37 without PIU.

While our meta-analysis revealed a clear pattern that individuals with ASD/high levels of autistic traits display an excessive and problematic use of the internet, there appears to be one exception: people high on the spectrum are less attracted to digital media that aim to facilitate social interaction, communication, and the sharing of personal information. For example, in a study by Durkin et al. (2010), a group of 35 adolescents with Asperger Syndrome (which was a specific subtype of ASD in previous classification systems; e.g., American Psychiatric Association, 1994) were compared to a matched control group of 35 typically developing adolescents with regard to their cell phone use. It was found that adolescents with Asperger Syndrome were generally less engaged with cell phones. When they did use one, they were less likely to use it for social interaction, such as contacting peers, and more likely to use its non-communicative features, like playing games, than subjects in the control group. Other studies have indicated that people with ASD are also less involved in prototypical social media such as Facebook, Snapchat, TikTok, and Instagram (e.g., Cardillo et al., 2025; Suzuki et al., 2021). Our meta-analysis—which synthesized all the research findings on this topic (Muris et al., 2025)—revealed a negative effect size of − 0.28, signifying that in general, people with ASD/high levels of autistic traits are less inclined to participate in social media.

## Case Description

This case vignette is drawn from a real clinical case. Identifying details have been altered to protect patient confidentiality.

Sebastian is an 18-year-old young man who has been diagnosed with autism spectrum disorder (ASD). As the oldest child of a German expat family, he attends an international school in The Netherlands where he is in pre-university education. Being in his senior year, his grades are largely in keeping with his cognitive capabilities (his verbal IQ on the Wechsler test was as high as 142, with all other factors being in the average to above average range). In social situations, he is shy and reserved but once he knows people better, he can become more communicative—though in an odd and rather unusual way. At the beginning of high school, he was more of a loner and at that time he also became victim of verbal and physical bullying. After an intervention of the school, this bullying stopped and his position in class gradually improved. During the last couple of years, he has even connected with some like-minded folk. However, he has no real friends and does not have the urge to meet up with others after school. Instead, he prefers to spend all of his free time on the internet. He has access to a smartphone, a laptop, and a desktop computer, and so there is plenty of opportunity to go online. Preferably, he stays in his bedroom to play a game for fun (“to get some dopamine,” in his words) or listen to a podcast or watch some YouTube videos on a topic of his interest (“to learn more and become smart”). He dislikes typical social media such as Facebook, Instagram, and TikTok because he has no intention to share any personal information and is “not interested in the superficial nonsense exhibited by others.” However, he is constantly using the chat widget when gaming to interact and communicate with the other players (“mostly about what is happening in the game but also about other more serious topics”) and occasionally also engages in a captivating discussion on the Reddit forum. Sebastian does not think that his use of the internet is problematic, but he frankly admits: “Although I am quite good at multi-tasking, the online distraction while doing my homework may have caused me to underperform in some classes.” His self-reported score on Young’s (1998) Internet Addiction Test (IAT) is 52, indicating a moderate level of problematic internet use. Sebastian’s mother has a somewhat different perspective. She provides a total IAT score of 81, which reflects a severe dependence on the internet and describes her son as “a very digital person who is always ‘connected’ and who becomes moody and irritable when he has no opportunity to go online.” During family gatherings, dinners, and even when doing chores in the house (e.g., cleaning or cooking), “there is always a screen in front of him.” As a result, he is not communicative and barely interacts with others. She has given up to correct this behavior as previous attempts have often resulted in huge conflicts and a lot of emotional turmoil in Sebastian, but her main worry is whether her son will ever be a full participant in society.

## Purpose of the Present Paper

The case of Sebastian further illustrates that people with ASD can show a pattern of internet use that is quantitatively and qualitatively different from that displayed by neurotypical individuals. Moreover, statements such as “to get some dopamine” and “not interested in the superficial nonsense exhibited by others” give us some insight in his motives for playing online games and avoiding internet platforms with a strong social orientation. Thus, to gain a better understanding of the aberrant internet use in ASD, it seems important to explore motivational factors underlying the online behavior of persons with this neurodevelopmental disorder. In general, when looking at reasons for why people use internet-mediated digital media, three main types of motives have been suggested: (1) Social motives, implying a desire to gain some social benefit; (2) Coping motives, reflecting attempts to deal with adversity and associated negative emotions; and (3) Enhancement motives, having to do with the promotion of a positive affective state (Bischof-Kastner et al., 2014; Rosell et al., 2023). In this paper, we will first analyze the driving forces behind the internet use of individuals with ASD and discuss how each of the three above-mentioned motives is modulated by the specific characteristics of the disorder. Then, we will adopt a developmental psychopathology perspective that includes these motivational tendencies that may help to understand the deviating internet use of individuals with ASD. Finally, we discuss the role of parents as an important regulating factor that may help young people with ASD to navigate safely online thereby preventing them to become caught in the web of the (inter)net.

## ASD and Motives of Internet Use

### Social Motives

The social motives of human beings have been comprehensively described in Fiske’s (2019) BUC(k)ET model. BUC(k)ET is an acronym incorporating the five core motives for why people seek interaction and communication with others: (1) Belonging, which has to do with the subjective feeling of being accepted, respected, included, and supported by others within a specific social context (Allen et al., 2021); (2) Understanding, which pertains to the strong drive of human beings to make sense of their world, share this with others, and have expectations fulfilled (Steger & Kashdan, 2007); (3) Control, referring to the ability to effectively initiate and manage social situations (i.e., social self-efficacy), which eventually facilitates the achievement of goals (Bandura, 1997); (4) Enhancing self, which is concerned with the inclination of people to think positively about themselves and to receive positive evaluations from others, thereby boosting their self-esteem (Alicke & Sedikides, 2011); and (5) Trust, which alludes to the inclination to establish mutually secure and caring relationships with others (Simpson & Vieth, 2021). Each of these motives also seems highly relevant for understanding people’s use of the internet for social purposes.

When discussing social motives in people with ASD, it is certainly relevant to refer to the social motivation theory of autism (Chevallier et al., 2012). This theory proposes that ASD is associated with diminished social motivation, which is manifested by less social orienting (Hedger et al., 2020), lower sensitivity for social reward (Bottini, 2018), and reduced use of social maintaining strategies (Short & Vital, 2021), all biologically mediated by dysfunctioning of the orbitofrontal-striatal-amygdala circuitry and dysregulations of certain neuropeptides and neurotransmitters (Bachevalier & Loveland, 2006). The straightforward application of the social motivation theory to the internet use of individuals with ASD would imply that social motives for going online are less relevant and less important for this population, and this might underpin the reduced social media use in ASD as found in our meta-analysis (Muris et al., 2025). However, questions have been raised regarding the validity of the social motivation theory of autism. For example, based on an analysis of testimonies of individuals with autism, Jaswal and Akhtar (2019) concluded that the primary assumption that people with ASD are socially uninterested is untenable as they often use other “unconventional—even idiosyncratic—ways [to] express their social interest” (p. 12). Examples of these include the excessive use of special interests as a ‘social bridge,’ proximity seeking without using verbal communication, mimicking or repeating phrases from films or books to relate socially, and showing affection by touching an object that the other person is using.

Indeed, the psychological literature reveals a perhaps more nuanced picture regarding each of the social motives described in the BUC(k)ET model. With respect to ‘Belonging,’ people with ASD may have a stronger need to be on their own (Kanner, 1943; but see Deckers et al., 2017), but there is also evidence indicating that they can identify themselves with social groups and that successful engagement with such groups promotes their well-being (Maitland et al., 2021). In terms of ‘Understanding,’ due to impairments in social cognition (Leekam, 2016), a weak central coherence (Happé & Frith, 2006), and detail-focused information processing (Van de Cruys et al., 2014), individuals with ASD may find it difficult to comprehend the complexities of their social and non-social surroundings. This does not mean, however, that they do not have the desire to obtain a better grasp of the external world thereby enhancing its predictability (Hickey et al., 2018). As for ‘Control,’ people with ASD display clear social cognition and social skills deficits (Baron-Cohen, 2001; Sasson et al., 2013b), which are reflected in the prototypical impairments in social communication and interaction. Given these difficulties, it is logical that they are also likely to suffer from social anxiety and consider themselves as less competent to handle real-life social situations (Spain et al., 2018). In terms of ‘Enhancing self,’ persons with ASD have been consistently found to report low levels of self-esteem (Van der Cruijsen & Boyer, 2021), which is probably due to the pervasive nature of this disorder that has a negative impact on various domains of life, resulting in an unfavorable comparison with others (McCauley et al., 2019). Finally, regarding ‘Trust,’ evidence suggests that individuals with ASD engage in similar processes of bonding to their parents (Taylor et al., 2008; Teague et al., 2017) and developing friendships with peers (Black et al., 2024) as their typically developing counterparts. However, it is also true that they often experience challenges and frequently fail to achieve such secure and caring relationships.

In conclusion, the core social motives also seem to apply to people with ASD, but prototypical social problems and peculiarities seem to obstruct them to actualize these motives and show normative socially skilled behavior (Jaswal & Akhtar, 2019). The internet provides individuals with ASD with a viable alternative for socializing and interacting with others (Leung et al., 2023). As noted by Gillespie-Lynch et al. (2014), participants with ASD indicated that online communication was easier to comprehend and control as compared to real-life communication (see also Poulain et al., 2024). In addition, the internet gives them access to other people high on the autism spectrum and provides them with an opportunity to express their true selves. It is important to note that the online communication of individuals with ASD does not always take place on the established social media platforms. Hughes and Nguyen (2024) noted that autistic adolescents playing video games frequently chat with other players, providing them with an option to communicate with others and to build friendships. Furthermore, in case they visit online social media, people with ASD tend to employ camouflaging (i.e., strategies to minimize the visibility of autistic traits; Cook et al., 2021) and often adopt a passive attitude (Jedrzejewska & Dewey, 2022), probably to avoid the risk of getting involved in unpleasant social interactions (e.g., cyberbullying; Holfeld et al., 2019; Triantafyllopoulou et al., 2022). Meanwhile, those who do succeed in using the internet effectively for establishing social connections—while evading the obvious ‘booby traps’ of this online technology—will satisfy existing social needs and ultimately develop a better general sense of well-being (Chan et al., 2023).

### Coping Motives

People with ASD have many challenges and difficulties in life, causing significant amounts of stress (Bishop-Fitzpatrick et al., 2017). A meta-analysis by Van Heijst and Geurts (2015) showed that across the entire lifespan, individuals with autism clearly have a lower quality of life than individuals without this neurodevelopmental disorder (see also Evers et al., 2022). This is mainly due to daily hassles and negative experiences associated with socio-emotional difficulties relating to lack of friendships (Black et al., 2024), loneliness (Grace et al., 2022), bullying (Humphrey & Hebron, 2015), social exclusion (Sasson et al., 2017), school problems (Horgan et al., 2023), employment barriers (Solomon, 2020), mental health issues (e.g., anxiety and mood disorders: Hymas et al., 2024; comorbid neurodevelopmental disorders: Khachadourian et al., 2023), and sensory sensitivities and rigidity (Leekam et al., 2011) that so many people with autism report to experience on a day-to-day basis.

The study of coping is highly relevant in this context. Coping refers to the ability to manage stressful or adverse conditions (Carver, 2019) and includes a variety of strategies, some of which are focused on efforts to resolve the source of stress (i.e., problem-focused coping; problem-solving, taking direct action) and others that are concerned with efforts to relieve one’s emotions (i.e., emotion-focused coping; e.g., distraction, avoidance, and withdrawal; Lazarus & Folkman, 1984). Research on how autistic people deal with problems is sparse, but in general individuals with autism are less capable of coping with the stress of everyday life as compared to their typically developing counterparts (Hirvikoski & Blomqvist, 2015). Furthermore, people with ASD often use emotion-focused coping strategies, which enable them to disengage from the stressor or the associated negative emotions (Corbett et al., 2021). This has probably to do with the fact that many stressors in the life of autistic people are quite complex and not easy to control, and as such require a planned long-term solution.

For example, feelings of loneliness may arise from various factors including social deficits and peculiarities, social anxiety, depression, negative learning experiences, and lack of friendships (Grace et al., 2022), which might be difficult to handle all at once. In a longitudinal study by Schiltz et al. (2024), a sample of 114 persons with ASD and related neurodevelopmental psychopathology was followed throughout their lives, beginning at age 9 and followed twice at age 19 and age 25. Many autistic participants reported to experience loneliness and indicated that these feelings increased when they became older. For example, at the age of 25, more than one third indicated loneliness as a major problem. When looking at how the individuals with autism dealt with loneliness, two main coping strategies emerged. The first one was used by 34% of the participants and was labeled as ‘instrumental action,’ a problem-focused strategy consisting of attempts to actively seek social contact with others. The second strategy of ‘behavioral distraction’ was even more often employed (50%): this strategy involved emotion regulation by engaging in some other pleasurable activity (e.g., watching TV, reading book). Interestingly, with increasing age, behavioral distraction by means of activities on electronic devices became more and more important (age 9: 9%, age 19: 57%, and age 25: 64%).

Taken together, given the pervasive nature of ASD, affecting so many domains of daily functioning, negative feelings of stress, anxiety, depression, and loneliness are common in these neurodivergent individuals. The internet offers a convenient medium to handle this emotional turmoil, offering persons an alternative way of escapism from the burdensome problems of the real life (Jouhki et al., 2022). In particular, online activities such as gaming and watching YouTube movies seem to be ideal for the purpose of managing negative emotions (Pyszkowska et al., 2023). There is a tendency to consider this type of emotion-focused coping as maladaptive (Taylor & Stanton, 2007), and there are indeed studies demonstrating that this type of avoidant and disengaging strategies is also less effective and eventually associated with less favorable outcomes in people with ASD (Khor et al., 2014; Muniandy et al., 2023). However, the effectiveness of coping strategies is dependent on context-related factors (Folkman & Moskowitz, 2004), and it is conceivable that there are circumstances in which the use of avoidance and other emotion-focused strategies is quite appropriate for an autistic individual (Mazefsky et al., 2013). Moreover, the internet not only serves to provide occasion for avoidance and escape but can also be used for more problem-focused purposes. For example, a person with ASD can seek social support and gain a sense of belonging by visiting an online forum for autistic peers (Caldwell-Harris et al., 2023), or use social media as a feasible possibility to get some kind of contact with others (Hudson et al., 2023) especially given its reduced complexity compared to face-to-face interactions (Van Schalkwyk et al., 2017).

### Enhancement Motives

Enhancement motives are concerned with intentions to derive a positive feeling from some activity. In the case of internet use, this pertains to the pleasure and entertainment obtained by engaging in online activities (Teo et al., 1999). This type of motives has been shown to be a good predictor of PIU in general (Bischof-Kastner et al., 2014; Kim & Haridakis, 2009; Rosell et al., 2023) and thus may also be particularly relevant for understanding the excessive use of the internet in people with ASD.

Research has shown that individuals with ASD have a natural affinity for technology and using digital media (Valencia et al., 2019). This probably has to do with autistic persons’ tendency toward systemizing, which can be defined as “the drive to analyze or construct systems” (Baron-Cohen, 2009) and has been associated with a preference for the highly predictable and objective world of technology and computers (Baron-Cohen, 2012). There is some evidence showing that the high digital media use of people with ASD is at least to some extent carried by positive emotions. For instance, Cardy et al. (2023) noted that according to the parents of young people with autism, enjoyment was the most important feeling (reported by 83%) associated with the use of technological devices such as the computer, tablet, and smartphone.

Another reason why persons with ASD experience online activities as pleasurable may have to do with their desire for solitude. This may be difficult to reconcile with findings on reported feelings of loneliness (Grace et al., 2022), but there are tentative indications that autistic individuals occasionally also have a strong need for being on their own (Bauminger & Kasari, 2000; Kasari & Sterling, 2014). Seeking seclusion with the computer and immersing oneself in the non-social digital world might be a suitable way to achieve this desire for aloneness.

The presence of restricted and unusual interests and behaviors is also a defining feature of ASD (American Psychiatric Association, 2022). Coutelle et al. (2022) conducted a review to analyze the vehicle behind the excessive video gaming of people with ASD. These scholars concluded that there is sufficient evidence to view the problematic gaming in this population as a type of addiction, but they also noted that the role of restricted interests cannot be excluded. For instance, the video game itself could be chosen because it compliments an already existing obsessive interest (Durkin, 2010). Moreover, outside of the context of gaming, the internet offers a wide range of options (e.g., discussion forums, YouTube, Wikipedia) to further cultivate a personal interest and greatly expand one’s knowledge about a particular topic. In line with this, in a non-clinical sample of undergraduates, Shane-Simpson et al. (2016) found that restrictive interests and behaviors were a better predictor of PIU than the social symptoms of autism, and that this relation was mainly driven by information seeking activities.

A special type of restricted interests and behaviors has to do with sensory reactivity. Some autistic individuals display an aberrant sensitivity to sensory input or a fascination in the sensory aspects of their environment (Robertson & Baron-Cohen, 2017). To characterize the restricted interests and behaviors in people with ASD, Uljarevic et al. (2022) interviewed the caregivers of 237 autistic children and adolescents about the frequency and type of interests in their offspring. Seventy-five percent of the youth had at least one special interest and 50% even had multiple special interests. Most importantly for the present discussion, most interests were sensory-based and mainly related to the visual modality. Lane and Radesky (2019) took this observation one step further by arguing that autistic individuals may prefer processing information in the visual modality, which could make the use of digital media even more appealing and intuitive for them.

The reinforcing nature of video games and other digital media is a final consideration when discussing the enhancing motives for using the internet. While the processing of rewards seems to be largely preserved in autistic individuals (Matyjek et al., 2023), their executive functions can be impaired (Hill, 2004), making them prone to develop difficulties with controlling or regulating their behavior. When managing stimuli and situations with high reinforcing value, this can cause problems, and in the case of PIU, gaming and watching online pornography seem to be especially relevant (Kim et al., 2020). Gaming can give a sense of power and agency, enabling autistic persons to demonstrate their ability and competences without the help of others (Pavlopoulou et al., 2022), while viewing porn may satisfy a need for intimacy which for autistic people is difficult to achieve in everyday life (Byers & Nichols, 2019).

According to Thorndike’s (1927) law of effect, behavior that is followed by a positive consequence is more likely to be repeated in the future. When applying this reinforcement principle to the enhancement motives for the use of internet, it is obvious that autistic individuals like to engage in online activities because they are attracted to and enjoy dealing with computers and other electronic devices. Furthermore, the digital world may offer them a good opportunity to satisfy their need to be alone or search for information and/or seek out activities in keeping with their specific (restricted and unusual) interests or stimulate the visual sensory modality (McGhee Hassrick et al., 2021).

## Developmental Psychopathology Model

**Fig. 1:**
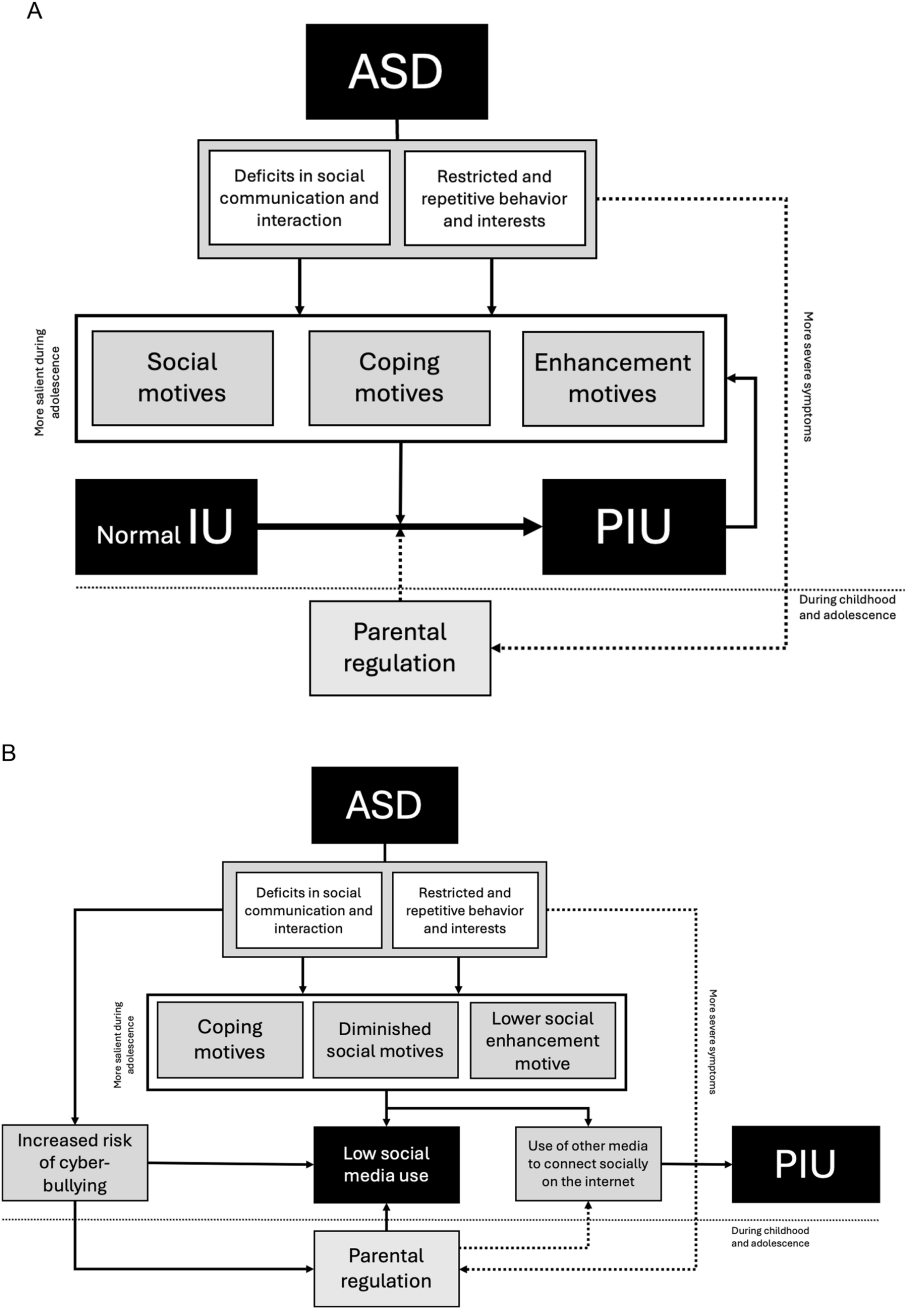
Motivational developmental psychopathology model of PIU (panel **A**) and (low) social media use (panel **B**) in ASD, including the role of parental regulation during childhood and adolescence. Solid arrows indicate a positive influence, and dashed arrows indicate a negative influence. *ASD* Autism Spectrum Disorder, *PIU* Problematic Internet Use

The above-described motivational analysis forms the basis for our developmental psychopathology model of atypical internet use in ASD. As can be seen in panel A of Fig. 1, the prototypical characteristics of this neurodevelopmental disorder fuel the three motives for engaging in online activities. Deficits in social communication and interaction may promote the drive to use the internet for social motives (as it provides a viable way to socialize and interact with others) but also prompt employment of the internet for coping motives (as it offers opportunities to forget the concerns of everyday life and deal with negative emotions). In a similar vein, the restrictive and repetitive behaviors and interests (RRBIs) may enhance the employment of the internet as a strategy for coping with overstimulation or as a way for seeking preferred sensory stimulation and satisfying the thirst for knowledge on specific, idiosyncratic topics of interest (Coutelle et al., 2022). In other words, the key clinical features of ASD prompt the motives for going online, mostly resulting in heightened levels of internet use and an increased chance that a normal deployment of the internet will develop into PIU.

Many internet-mediated digital media are built in such a way that they incorporate addictive features that aim to prolong the time spent on these services (Montag et al., 2019). People who have strong motives to use the internet, such as individuals with ASD, are especially prone to fall victim to PIU. This can happen at any point during a person’s life, but adolescence seems to be particularly relevant in this regard because of the marked biopsychosocial changes that occur during this developmental stage, which make each of the above-mentioned motives for using the internet (i.e., social, coping, and enhancement) even more salient. In their gradual transition to adulthood, adolescents face several important developmental hurdles (e.g., the development of autonomy, establishing connection with peers, and the formation of identity), which have to be taken during a period of extensive brain development. Research suggests that the hormone-induced brain maturation and its behavioral sequelae are implicated in the social hassles, emotional turmoil, and heightened reward sensitivity of young people (Crone & Dahl, 2012; Walker et al., 2017), and some scholars have linked these to the increased digital media use typically noted during this developmental stage (Crone & Konijn, 2018). There are also clear indications that the brain of people with ASD develops in a different way (compared to typically developing persons), leading to deviating activation and regulation processes (Müller & Reiter, 2019) and posing greater challenges to the management of social, emotionally salient, and rewarding cues (Corbett & Simon, 2014) that are often occurring within the context of the internet.

Regarding the use of social media in people with ASD, our model is also relevant, although the motivational mechanisms seem to be operating in a somewhat different way (Fig. 1, panel B). To begin with, in case deficits in social communication and interaction are so severe that connecting to others via social media elicit considerable discomfort, the person will avoid this type of internet activities (coping motive). Furthermore, people with ASD may generally have less interest in connecting to people (Chevallier et al., 2012) and/or tend to prefer interaction and communication with others in a less intense, idiosyncratic way (Jaswal & Akhtar, 2019), which may also diminish the person’s motivation to access the prototypical social media (social motive). If they do wish social contact, they may prefer to use other digital media, such as the chat function in online game environments (Bowman et al., 2022), which could, in turn, contribute to the development of PIU. Finally, RRBIs-related issues such as social overstimulation—which may prompt a strong wish for aloneness, and an increased interest in non-social stimuli—which continuously competes with the attention for social stimuli (Clements et al., 2018)—further lower the social media use of people with ASD (enhancement motive). Apart from these motivation-based mechanisms, people with ASD also run the risk to become victim of cyberbullying or sexual harassment (Hu et al., 2019; Macmillan et al., 2022), and obviously, this might be another reason for being more reluctant to visit social media platforms.

While the harmful effects of PIU are clearly apparent (e.g., McGhee Hassrick et al., 2021), it can be debated whether the lowered levels of social media use in individuals with ASD should be qualified as ‘problematic’ as not using Twitter, Instagram, and Facebook does not seem to be the end of the world. For example, Pennington (2020) argued that stepping away from mainstream social media may reflect a deliberate choice or preference, potentially leading to benefits such as more meaningful interpersonal interactions and reduced harmful social comparisons. Meanwhile, there is some evidence indicating that young people with ASD who do visit social media sites report a better quality of friendships than youngsters with ASD who do not visit these platforms (Costescu et al., 2024; Kuo et al., 2014; Van Schalkwyk et al., 2017), which echoes the notion that social media may act as a social lubricant facilitating the interaction and communication with others in real life (Leonardi & Meyer, 2015).

Developmental psychopathology models assume that—besides biologically determined vulnerability—environmental influences also play a role in the formation of abnormal behaviors (Cicchetti, 2016). In the case of PIU and social media use, parenting seems to be a particularly relevant environmental factor. Especially during childhood, parents have an important say in the type, frequency, and duration of the online activities of their children, although parental restrictions typically subside during the adolescent years and youngsters are increasingly required to rely on themselves to regulate their use of the internet. Given its potential role during the first two decades of life, the next section zooms in on the role of parental regulation of their offspring’s employment of internet-mediated digital media in more detail.

## Parental Regulation of Internet Use

Contemporary parents face the challenging task to help their offspring deal effectively with electronic devices and navigate them through the complexity of the digital world. Exposure to inappropriate content, cyberbullying, and excessive screen time (with all of its associated negative consequences) oftentimes elicit concerns among parents (Choy et al., 2024). Digital parenting is a term dubbed to refer to all parental efforts and practices that have the purpose to understand, support, and regulate young people’s activities in digital environments (see Modecki et al., 2022). This regulation encompasses strategies that help children and adolescents to become independent and responsible users of internet-mediated devices (by means of instruction, discussion, encouragement, and modeling), while ensuring that offspring do not engage in unsafe online activities and keep a proper balance between online and offline time. Examples of the latter strategies include restriction and rule setting (e.g., limiting time that can be spent online or access to certain platforms, and monitoring). Apart from specific digital parenting strategies, more general parenting and family factors also play a role in the regulation of offspring’s internet use.

A systematic review focusing on the relation between general parenting behaviors and PIU (Nielsen et al., 2020) has demonstrated that positive parenting (e.g., emotionally warm, authoritative, and supportive) and positive family dynamics (e.g., secure attachment, family cohesion) are associated with lower levels of PIU, while negative parenting (e.g., rejective, authoritarian, and critical) and negative family dynamics (e.g., child abuse, poor family relations) are accompanied by high levels of PIU, with most of the observed effect sizes being in small to medium range. Regarding the effects of internet-specific parenting strategies, the evidence is more mixed and inconclusive. For example, Vossen et al. (2024) found that parental attempts to restrict the internet use of their offspring—by setting clear rules about when, where, and for how long the internet may be accessed—have a preventive positive effect, thereby reducing the risk of developing PIU. However, when parental restriction is conducted in a more reactive way, for example, when parents intervene with ongoing internet use of their offspring by ordering them to stop in a blanket manner, the effects tend to be negative, eventually resulting in higher levels of online behavior (Koning et al., 2018).

Research has indicated that parents of children and adolescents with ASD experience higher levels of parenting stress than parents of children and adolescents without this neurodevelopmental disorder (Hayes & Watson, 2013). These heightened stress levels can be mainly ascribed to the severity of autistic symptoms (i.e., communication and interaction problems and repetitive behaviors and interests) and associated challenging behaviors (such as aggressive and self-injurious behaviors) as displayed by their offspring (Argumedes et al., 2018), which would undermine the ability to complete parenting tasks and have a negative impact on the quality of parenting (Ooi et al., 2016). The presence of comorbid externalizing problems might be another challenge that parents of autistic youth have to deal with. For example, a study by McStay et al. (2014) has indicated that after controlling for autism severity, concurrent symptoms of disruptive behavior disorder and ADHD were significantly associated with higher levels of parental distress.

Basically, parents of young people with ASD appear to rely on similar strategies to regulate the internet use of their offspring as parents of typically developing offspring (Mayer et al., 2024). However, the high stress levels experienced by these parents may have an additional effect on how they manage the screen time of their autistic children and adolescents. This issue was illustrated recently in an interesting study by Bozoglan and Kumar (2022) who examined the relations between negative (harshness, permissiveness, and physical discipline) and positive (proactivity, positive reinforcement, warmth, and support) parenting and parental stress on the one hand, and autistic children’s levels of excessive internet use on the other hand. It was found that negative parenting (but not positive parenting) and parental stress each explained a unique and significant proportion of the variance in children’s PIU scores. It is likely that parents of autistic youth allow their offspring to use digital media as a soothing strategy (Menezes et al., 2024) and also rely on this approach to keep the household quiet and to calm themselves (Lane & Radesky, 2019). The risk of this approach is that parent and child enter a coercive process: the child is showing difficult behavior, followed by the parent’s reaction allowing the child to use a digital device because this results in a decrease of difficult behavior in the child and hence a reduction of parental stress, at least in the moment. Through the mechanism of negative reinforcement, however, this will eventually lead to increased screen times and even use of digital media in unwanted situations (e.g., when doing homework or in bed; Engelhardt & Mazurek, 2014; Mazurek et al., 2016).

Few studies have been conducted on parental regulation of the social media use in young people with ASD. However, (the threat of) cyberbullying and other forms of harassment encourage parents to caution their (socially vulnerable) autistic children about these events and take protective steps to prevent them from becoming victims of such negative experiences. A review by Elsaesser et al. (2017) found that parents generally use a combination of monitoring, mediation, and restrictive strategies to prevent unwanted social media contacts and cyberbullying among their offspring. An evaluation of these efforts showed that cooperative strategies—such as open discussions about internet use and mutual agreements on which platforms to use—produce the best outcomes, especially when implemented in a warm and open manner. This approach, conducted along the principles of the Collaborative and Proactive Solutions model (Greene & Winkler, 2019), might increase the likelihood that the child will feel comfortable sharing his online activities and experiences with the parents. There is some evidence that this conclusion may also apply to parents of young people with ASD (Tschida et al., 2021; Wright, 2017), but further research is clearly needed to address this possibility.

## Discussion

Taken together, our developmental psychopathology model describes three types of key motives (i.e., social, coping, and enhancement) that push people toward digital devices to engage in various types of internet-mediated activities (e.g., gaming, viewing YouTube clips). There are good reasons to assume that these motives are mostly stronger in persons with ASD, in particular during the developmental stage of adolescence when young people face a number of significant biopsychosocial challenges (Goldstein, 2020) and—because of reduced parental restrictions—are increasingly thrown back on own abilities to control their internet use. There are also indications that in cases where the young person displays more severe ASD symptomatology, parental regulation of internet use may already have diminished at a much earlier age. Note also that there is a positive feedback loop between PIU and motives for using the internet: excessive online activities may intensify social and emotional problems (promoting social and coping motives) but may also yield (short-term) reward (promoting the enhancement motive; Davis, 2001).

The model describes how (young) people with ASD gradually become “caught in the web of the net” and increasingly engage in all kinds of internet activities. As also pointed out by our meta-analysis in Part I (Muris et al., 2025), the use of social media seems to be the one exception: persons with ASD spend less time on prototypical social media platforms. This finding can also be explained by the same three motives outlined in our developmental psychopathology model, although their operation may differ. More specifically, people with ASD may have a diminished drive to engage socially than neurotypical people (Chevallier et al., 2012), tend to avoid social media because interactions with others might be too challenging (Wang et al., 2020), or prefer non-social activities that are in keeping with their restricted interests rather than connecting with others on social platforms (Kuo et al., 2014). Meanwhile, as has been mentioned earlier, one should be aware of the fact that people with ASD may have idiosyncratic ways of seeking social contact. For example, excessive gaming may incorporate high levels of social communication and interaction by the frequent use of the chat facility (Bowman et al., 2022), but this possibility has remained largely unexamined in past research.

To test the tenets of our motivation-based model of internet use and in specific to examine the involved underlying motives, we suggest two possible research methods. The first possibility would be to conduct studies that include global self-reports to measure motives of online activities. An example is the Questionnaire of Motives of Internet Use (Rosell et al., 2023), which measures five types of motives of people for going online: social motives (obtaining some social benefit), coping motives (dealing with negative emotions and distress), enhancement motives (achieving a positive psychological state), conformity motives (which are also social but more negatively driven, i.e., avoiding exclusion or disapproval from others), and utility motives (going online for practical reasons related to work, education, paying bills, and information seeking). Another scale that is more relevant for social media use is Tuck and Thompson’s (2024) Social Media Use Scale, which maps active, passive, social, and non-social motivations for visiting social networking platforms. These scales could be employed in cross-sectional and prospective research to examine the relations between various motives and online behaviors and evaluate their relative contributions in the PIU/social media of people with ASD. Another option would be to use Ecological Momentary Assessment (EMA), a method that entails repeatedly collecting data on participants' current behaviors, emotions, thoughts, and experiences in real time (Shiffman et al., 2008). The use of EMA would enable researchers to study the micro-processes that influence the online actions of people with ASD (and typically developing persons) in a real-world context, and hence give a detailed picture of the underlying motives of online behavior of persons high on the autism spectrum. Both research approaches could also explore gender differences in the motivations behind internet use, the manifestation of PIU and social media usage, and potential online camouflaging behaviors of autistic individuals (Jedrzejewska & Dewey, 2022; Mari et al., 2023; Teo & Lim, 2000).

While our model incorporates several key factors relevant to PIU and social media behavior among individuals with ASD, it is not without limitations. Notably, the model focuses on neurodivergent individuals with typical intellectual functioning. However, it is important to recognize that many people with ASD also have intellectual disabilities (Matson & Shoemaker, 2009), which can significantly affect how—and whether—they access the internet (Chadwick et al., 2013). This, in turn, has implications for the generalizability and validity of the model. Additionally, although internet use, PIU, and social media engagement are global phenomena, substantial cultural differences may influence the online behavior of autistic individuals. For instance, Kim and Ju (2019) observed that prototypical social media platforms are more widely used in individualistic societies than in collectivistic ones, highlighting the importance of cultural context in interpreting online behavior.

The developmental psychopathology model of PIU in ASD as described in this paper may also have implications for attempts that aim to help people with ASD navigate in a more healthy and positive way on the internet. To begin with, treatment of PIU has mainly focused on cognitive-behavioral therapy (CBT; Roberts et al., 2022), which primarily targets the coping motive underlying excessive online behavior. This to some extent makes sense as anxiety and depression are often implicated in PIU and so it seems logical to address these aspects of negative emotionality. Although CBT-based treatments have proven to be effective for treating PIU in typically developing persons (Winkler et al., 2013), it may well be the case that when treating individuals with ASD, interventions need to include additional elements in order to deal adequately with specific social and enhancement motives.

Furthermore, for young people with ASD, interventions might be helpful that target parents’ way of dealing with their offspring’s internet use (Good & Fang, 2015). There is tentative evidence that a parent-based training focusing on the enhancement of communication and interaction between autistic preschool children and their parents, while setting clear boundaries to the use of digital devices, yields positive results (Sadeghi et al., 2022; Tschida et al., 2021). However, more studies are needed to examine the effects of parent interventions that target the prevention and diminishment of excessive internet use in young people with ASD during the more challenging stage of adolescence. Finally, notwithstanding potential benefits of social media (Leonardi & Meyer, 2015) that could be profitable to people with ASD as well (Kuo et al., 2014; Van Schalkwyk et al., 2017), current popular platforms may be difficult to handle for some autistic individuals given the nuances of social interactions and deceptive tactics of malicious users. Some scholars have pleaded for re-designing social media in such a way that they are more inclusive for persons who are high on the autism spectrum and offering special online training programs to promote the safety on social platforms for individuals with ASD (Wang et al., 2020).

In conclusion, the present theoretical article points out that the online behavior of people with ASD or high levels of autistic traits is a topic with great clinical potential. The case of Sebastian is certainly not an exception. Although he was still functioning quite well, there are many more patients with ASD who—because of their excessive internet use—become blocked in their development toward adulthood. Therefore, more research is needed that uses refined methodology to reveal the exact motives and mechanisms underlying the aberrant use of internet-mediated digital media of individuals high on the autistic spectrum. In the meantime, clinicians should also pay more attention to the (problematic) internet use of persons with ASD as this would give them a more complete picture of dynamics of their clients’ problems.

## Data Availability

No datasets were generated or analysed during the current study.
